# Informal Near-Peer Teaching Effects on Medical Student Pre-Clinical Learning

**DOI:** 10.1007/s40670-026-02722-7

**Published:** 2026-04-20

**Authors:** Rigel P. Hall, Michael M. Fontana, Katherine L. Frei, Claudia Mares-Gonzalez

**Affiliations:** Creighton University School of Medicine, Phoenix, AZ USA

**Keywords:** Near-peer teaching, Health professions education, Peer-to-peer teaching, Preclinical curriculum, Academic performance

## Abstract

**Background/Purpose:**

Informal Near-Peer Teaching (NPT), where a student at least one year senior to another instructs without faculty intervention, has long been implemented in medical curricula. However, there is a further need for formal evaluation of the effectiveness of NPT. As with traditional curriculum, continuous, evidence-based assessment and improvement are essential to maintaining excellence in medical education. The purpose of this study is to evaluate the effects of informal NPT sessions on preclinical medical student learning.

**Methods:**

Student performance data from three pre-clerkship cohorts were analyzed and stratified by NPT exposure. Cohort performance was compared, and regression analyses assessed associations between NPT attendance and assessment outcomes.

**Results:**

Across two preclinical blocks, students who received NPT—refined over two years—consistently outperformed those without exposure on multiple quizzes, final exams, and overall block grades. When examined separately by class year, results were mixed, with NPT exposed cohorts demonstrating improvement on some assessments and lower performance on others. When pooled, cohorts with any NPT exposure demonstrated significantly higher scores than those with no exposure. Regression analyses showed a positive, though not uniformly significant, association between NPT attendance and performance outcomes.

**Conclusion:**

Informal near-peer teaching was associated with improved performance in select assessments, particularly after two years of program development. These findings support the integration of NPT into preclinical curricula and highlight the importance of continued program refinement and evaluation.

## Introduction

Near-Peer Teaching (NPT) has been defined as a pedagogical approach in which students who are one year or more senior provide instruction, guidance, or mentorship to junior learners within the same educational program or discipline [1–7]. Because the structure of academic medicine inherently creates teams with members across the spectrum of training, this learning modality often arises informally. However, medical educators are increasingly acknowledging the value of NPT, and instituting formal programs to support and facilitate this learning method [8, 9]. Although formal data on the prevalence of NPT across medical schools are limited, the growing volume of recent literature on near-peer teaching indicates this approach is gaining popularity within medical education [8, 10–12].

Prior systematic reviews and meta-analyses suggest NPT has positive impacts on medical student learning, with comparable results in knowledge acquisition and practical skills development to faculty-led instruction [11, 13, 14]. NPT improves student testing scores and consistently earns high student satisfaction ratings due to approachability, relatability, and facilitation of a supportive learning environment [11, 13, 15]. This approach is not without its criticisms, as Bowyer and Shaw [16] identified a lack of training prerequisites, knowledge deficits of peer instructors, lack of content monitoring, and reliance on student generated study materials that may vary in accuracy, depth, and alignment with course objectives as key weaknesses of the NPT method. Other studies have noted NPT to be less effective in advanced, discipline-specific content [10, 13]. Altogether, the literature best supports the use of NPT as a complement to faculty-led teaching with positive impact on test performance [11, 13–17].

Creighton University School of Medicine (CUSOM) formally implemented NPT instruction through Supplemental Instruction (SI) sessions. These sessions are optional, weekly reviews for first year medical students (M1) led by second-year medical students (M2) that cover preclinical course material taught by faculty in the previous week. Although Creighton’s NPT program was formally implemented under the School of Medicine’s Office of Student Affairs, teaching material and resources are entirely student-sourced, and sessions have very minimal faculty oversight. In an attempt to mitigate some of the weaknesses of NPT identified by Bowyer and Shaw [16], SI leaders were selected from second-year medical students who exhibited strong academic performance in first-year blocks and had previous teaching experience, and SI teaching material was heavily based on lecture content delivered by School of Medicine faculty. The SI program was recently piloted; thus, only two classes have received these SI sessions for the entirety of the year. The novelty of SI at the Creighton Phoenix campus and the lack of previous formal evaluation of this program provided a valuable opportunity for assessment and evidence-based curriculum improvement. We hypothesized that the CUSOM cohorts who received SI sessions would exhibit better performance in weekly quizzes, exams, and final block grades.

## Methods

### Data Source/Study Population

This study was reviewed by the Creighton University Institutional Review Board and determined to be exempt under 45 CFR 46.104(d), as all analyzed data were de-identified. Student data from CUSOM’s Phoenix classes of 2026, 2027, and 2028 were collected to investigate the relationship between implementation of SI and academic performance. Individual quiz, exam, and final course grade data were collected from the Immunology and Musculoskeletal (MSK) blocks. Quizzes and exams for these blocks consisted solely of multiple-choice questions. For the Immunology block, each individual quiz accounted for 8% of the block grade (24% total) and the final exam accounted for 68%. The remaining 8% of the Immunology block grade consisted of Creighton’s case-base learning and team-based learning assessments. For the MSK block, each individual quiz (6 total) accounted for 2% of the block grade (12% total) and the final exam accounted for 48%. The MSK quizzes and final exam were a smaller weight of the block grade due to the inclusion of a separate anatomy practical. The remaining 40% of the MSK block grade included this anatomy practical assessment, and Creighton’s case-base learning and team-based learning assessments. The number of questions on the final exam in the Immunology Block for classes 2026, 2027, and 2028 were 72, 73, and 75 respectively. The number of questions on the final exam for the MSK Block for classes 2026, 2027, and 2028 were 92, 95, and 95 respectively. These blocks, defined as discrete study periods in medical education focused on a specific subject, were selected for their unique characteristics. The Immunology block represents an early-stage curricular transition from foundational material, introducing students to specialized content for which they typically have limited prior exposure. This minimizes potential confounding attributable to pre-existing knowledge. The Musculoskeletal block was chosen given its extended duration of eight weeks, which facilitates longitudinal assessment of student performance. Occurring later in the academic year, it additionally provides an opportunity to evaluate the impact of supplemental instruction once students have acclimated to the academic rigor and workload of medical school. Grading data was captured from 130 students in the class of 2026, 132 students in the class of 2027, and 130 students in the class of 2028. Students in the classes of 2027 and 2028 were exposed to NPT through the SI program, while students in the class of 2026 were not. Three quizzes and the block final in the Immunology block and 6 quizzes and the block final in the MSK block were analyzed. The shorter Immunology block includes fewer quizzes by design.

### Statistical Analysis

Two-tailed independent-samples student’s t-tests and simple linear regression models were conducted using IBM SPSS Statistics, version 28.0 (IBM Corp., Armonk, NY). Student’s t-tests were performed to compare sample means between groups based on receiving NPT through the SI program. The Class of 2026 served as a control, having never received SI instruction. The primary comparison was made between the Class of 2028 and the Class of 2026. By 2028, the supplemental instruction (SI) program had undergone two years of development, and instructor rotation had increased to a weekly schedule, cycling every three weeks, thereby making the observed effects more reflective of the program itself rather than individual instructor ability. Additionally, outcomes for the Class of 2027, the first cohort of M1 students to receive SI, were compared to those of the Class of 2026.

Secondary analyses were conducted within the Class of 2028 and Class of 2027 using regression analyses to evaluate a potential correlation between SI attendance and quiz scores. SI attendance was collected through a QR code survey that students were asked to complete when they attended a SI session, in person or online, and a deidentified total attendance was recorded for each session. This self-reported attendance data was used in the regression analyses with quiz scores.

## Results

In the Immunology block (Table 1), performance differed significantly across cohorts. Compared to the Class of 2026, the Class of 2028 demonstrated higher mean scores on Quiz 2 (*p* = 0.015), Quiz 3 (*p* = 0.019), the Final Exam (*p* = 0.011), and the overall Block Grade (*p* = 0.021). The Class of 2027, however, performed significantly lower on Quiz 1 (*p* = 0.0017), with borderline lower performance on Quiz 3 (*p* = 0.060) and the Final Exam (*p* = 0.087). When the Classes of 2028 and 2027 were pooled, scores remained significantly different from 2026 for Quiz 1 (lower, *p* = 0.0036) and Quiz 2 (higher, *p* = 0.022), while differences in later assessments were no longer significant.

**Table 1:** Student grade data from within the immunology preclinical block

	Immunology block performance		
	Class of 2028 *n* = 130 Mean (SD)	Class of 2027 *n* = 132 Mean (SD)	Class of 2026 *n* = 130 (Mean (SD)
Quiz 1	86.2 (9.8)	84.8 (9.2)	88.2 (8.1)**
Quiz 2	83.6 (7.5)*	82.9 (8.8)	81.3 (7.7)**
Quiz 3	83.5 (8.4)*	78.6 (8.9)	80.8 (9.9)
Final Exam	88.9(6.9)*	84.8 (9.3)	86.6 (7.6)
Block Grade	87.5 (6.4)*	84.7 (6.2)	85.6 (6.8)

*Indicates significance (*p*<0.05) in comparison to class of 2026 ** Indicates significance (*p*<0.05) in comparison to pooled data of 2027 and 2028

In the MSK block (Table 2), significant improvements were observed primarily in the Class of 2028. Compared to the Class of 2026, the Class of 2028 scored higher on Quiz 2 (*p* = 0.0002), Quiz 4 (*p* = 0.008), Quiz 6 (*p* = 0.0002), and the Final Exam (*p* = 0.023), though lower on Quiz 5 (*p* = 0.007). The Class of 2027 demonstrated significantly higher performance only for the Block Grade (*p* = 0.009), while other quiz and exam scores were not significantly different from the Class of 2026. Pooled analysis was conducted to assess whether combining both exposure cohorts revealed consistent patterns of improvement. When pooling the Classes of 2028 and 2027, performance was significantly higher than the Class of 2026 for Quiz 2 (*p* < 0.001), Quiz 4 (*p* = 0.015), Quiz 6 (*p* = 0.0036), the Final Exam (*p* = 0.038), and the Block Grade (*p* = 0.029).

**Table 2:** Student grade data from within the musculoskeletal preclinical block

	MSK Block performance		
	Class of 2028 *n* = 130 Mean (SD)	Class of 2027 *n* = 132 Mean (SD)	Class of 2026 *n* = 130 Mean (SD)
Quiz 1	89.2 (7.8)	87.6 (8.2)	87.5 (8.6)
Quiz 2	87.4 (8.5)*	89.4 (7.1)*	83.3 (8.7)**
Quiz 3	88.7 (8.8)	87.3 (8.5)	88.1 (8.3)
Quiz 4	88.0 (7.5)*	87.1 (8.7)	85.3 (8.7)**
Quiz 5	82.6 (10.1)*	85.5 (8.2)	85.9 (9.4)
Quiz 6	89.4 (8.0)*	87.0 (8.0)	85.4 (9.2)**
Final Exam	85.8 (6.0)*	85.1 (6.0)	84.0 (6.7)**
Block Grade	85.8 (5.8)	86.8 (5.6)*	84.9 (6.1)**

*Indicates significance (*p* < 0.05) in comparison to class of 2026 ** Indicates significance (*p* < 0.05) in comparison to pooled data of 2027 and 2028

Attendance-based analyses showed weak to moderate positive correlations between NPT attendance and performance, though none reached statistical significance (Table 4). In the Immunology block, correlation coefficients were 0.81 (*p* = 0.19) for the Class of 2028 and 0.91 (*p* = 0.21) for the Class of 2027. In the MSK block, correlations were minimal, with *r* = 0.03 (*p* = 0.96) for the Class of 2028 and *r* = 0.23 (*p* = 0.62) for the Class of 2027.

## Discussion

In analysis of this study, implementation of informal NPT through SI was associated with a neutral or positive impact on academic performance across several assessments and overall block grades in two preclinical blocks. Positive impact on student performance can be attributed to significant changes noted by increased scores on quizzes, final exams, and overall block grades. Since the data collected does not show significant impairments in student performance after NPT resources were implemented, NPT is associated with a neutral impact on student performance. Near-peer teaching (NPT), in which senior medical students provide instruction to junior peers without direct faculty oversight, has become increasingly integrated into medical curricula as a means of promoting collaboration, approachability, and learner engagement [1–3]. Despite its growing popularity, few studies have quantitatively evaluated the impact of informal NPT programs on objective student performance outcomes. The present study sought to address this gap by assessing the effect of Supplemental Instruction (SI)—an informal, student-led near-peer model—on preclinical academic achievement at the Creighton University School of Medicine – Phoenix campus.

The results suggest informal NPT through SI was associated with a neutral and/or positive impact on academic outcomes, particularly following two years of program refinement. This refinement included revising previous SI slides to enhance relevance, highlighting and annotating slides used in course lectures, adding student-authored and reputably sourced practice questions (First-Aid, Boards Vitals, AMBOSS, etc.) for high-yield topics, and providing additional recommended resources for the weekly content. The Class of 2028, which participated in a more developed iteration of SI, demonstrated significantly higher performance across multiple quizzes, final exams, and overall block grades compared with the pre-implementation Class of 2026. While the changes in scores may not reflect changes in traditional letter grades such as ‘A, B, or C…”, marginal increases in scores still have the potential to hold significance in student performance. Creighton University uses a grading system which includes honors, satisfactory, and unsatisfactory grades. These categories of grades are based on the percentage of students’ scores compared within the class across campuses, which indicates exceptional student performance in the top 10% earning an honors classification. In addition, marginal increases hold potential to positively impact students on the cusp of earning a satisfactory grade, a 70% in overall course average or higher. This is especially impactful as NPT has an association with neutral or positive impact on final exam grades, which holds the greatest weight of the students’ overall block grade. These findings align with prior research indicating that NPT can complement faculty-led instruction by providing additional opportunities for engagement, clarification, and repetition of complex material [11, 13, 14].

The mechanisms underlying these improvements likely include the value of peer congruence and the structure of SI itself. Senior students, having recently completed the same coursework, are uniquely positioned to identify high-yield topics, clarify difficult concepts, and model effective study strategies. This peer dynamic fosters an approachable learning environment where first-year students can comfortably ask questions and reinforce comprehension—factors known to enhance self-efficacy and confidence [18, 19]. While peer dynamics have been traditionally assumed to be in-person relationships, the SI model implemented at Creighton University School of Medicine (CUSOM-PHX) offers both in-person and virtual modes of resource material, improving access amongst students. The reported data in Table 3 is largely representative of in-person or simultaneous usage of SI sessions. However, SI materials, such as lecture Power Points, practice questions, and recordings are believed to be more widely used, greater than 88% of the class population, compared to what the in-person attendance data represents (see Limitations section, lines 264–267) (Table 4).

**Table 3:** Class of 2027 and 2028 student attendance data from within the Immunology and MSK blocks

Immunology Attendance		
Week	Student Attendance 2027	Student Attendance 2028
1	31	41
2	16	30
3	No data recorded	32
4	26	39
MSK Attendance		
Week	Student Attendance 2027	Student Attendance 2028
1	53	24
2	62	42
3	59	34
4	40	52
5	54	32
6	20	41
7	49	44

**Table 4:** Correlations between attendance and grade performance by academic year and block

	ImmunologyAttendance grade correlationr (*p*-value)	MusculoskeletalAttendance grade correlationr (*p*-value)
Class of 2028	0.81 (0.19)	0.03 (0.96)
Class of 2027	0.91 (.21)	0.23 (0.62)

The Musculoskeletal (MSK) block provides a particularly illustrative example of how near-peer instruction can positively impact student performance on weekly quizzes, final exam grades, and overall block grades. MSK is the longest and most content-dense block in the preclinical curriculum. MSK spans eight weeks and integrates an extensive range of educational modalities, including 52 faculty-led lectures, 16 four-hour anatomy laboratory sessions (with integrated team-based learning), eight case-based discussions, six quizzes, a laboratory practical, and a cumulative final examination. This volume and variety of material—covering anatomy, physiology, embryology, and pathology of the musculoskeletal and integumentary systems—can be challenging for early medical students to synthesize and retain. The positive impact of NPT can be seen by improved academic performance in the final exam grade and overall block grade when comparing pooled data from classes 2027 and 2028 to the class of 2026, which did not have access to SI. Specifically, for the MSK block which spans the greatest course length, with the largest amount of academic material, SI sessions could aid students in organization of overall course content, leading to improved performance on final exam and block grades cumulatively.

A potential explanation for the association between NPT implementation and improved test performance is that the Supplemental Instruction sessions may have helped students manage this cognitive load by condensing broad, complex content into focused, high-yield reviews aligned with ongoing coursework. Peer instructors utilized diverse pedagogical methods such as visual diagrams, practice questions, summary slides, and discussion-based problem solving to reinforce key themes. Furthermore, since these instructors had recently completed the same block, they could emphasize recurring exam topics and clarify nuances of clinically relevant anatomy and pathophysiology. This structure may have provided students with a framework for integrating information from lecture, laboratory, and case-based formats into cohesive conceptual understanding.

Moreover, SI’s informal and supportive environment potentially facilitates students’ iterative engagement with material rather than reactively revisiting difficult concepts weekly and building cumulative mastery throughout the block. Literature has shown that repetition and distributed practice of material improve students’ recall and retrieval of learned information, and thus overall academic performance [20, 21]. The benefits observed in MSK performance metrics, including higher quiz and final exam scores for the 2028 cohort, likely reflect the structured repetition and the metacognitive advantages inherent to near-peer instruction.

While the mechanisms of benefit were similar, the impact of NPT in the Immunology block underscores its value in subjects that are conceptually novel to first-year students. For many learners, Immunology represents their first in-depth exposure to molecular and cellular mechanisms of immune regulation. Peer-led SI sessions appeared to mitigate this learning curve by simplifying complex topics and guiding students through foundational principles with relatable explanations. The pattern of results—with improvements on several assessments but not uniformly—may reflect the varying degree to which SI content aligned with specific quiz emphases, complexity of topics, and how students engaged with materials across the block. Overall, the Class of 2028 demonstrated a positive performance across quizzes, the final exam, and the overall block grade compared with the pre-SI cohort.

Overall, NPT through SI is provided with the intention to support student learning and be an available resource to aid students who feel they need supplemental instruction to corroborate their learning. The classes who received SI sessions (2027 and 2028) were associated with a neutral or positive impact on their academic performance in comparison to the class of 2026 when NPT was not available.

## Limitations

Several limitations warrant discussion. While attendance data did not show a statistically significant correlation with exam performance, this likely reflects incomplete measurement of true engagement. Deidentified total attendance for each session was tracked through QR code sign-ins at in-person and online sessions; This data is self-reported and may not reflect students’ use of asynchronous materials such as recordings, slides, and shared study guides. However, follow-up attendance tracking conducted outside the formal study period—during a week in which access to course materials required attendance—demonstrated that 106 of 120 students accessed the SI slides. Although limited to a single week and occurring outside the primary study window, these findings, when considered alongside subjective student polling, suggest that SI sessions were highly utilized across cohorts, regardless of session attendance. Future studies should quantify total NPT utilization rather than attendance alone to better assess exposure to materials and overall SI content engagement. Additionally, attendance data was collected inconsistently throughout our study period, resulting in weeks with no recorded data. This makes within cohort analysis of attendance effects on academic performance difficult. Future studies should investigate whether attendance of SI sessions would result in significant differences in assessment outcomes within a cohort.

Second, inter-cohort comparisons risk confounding from differences in baseline academic preparedness or curricular evolution. However, review of institutional data revealed comparable incoming MCAT scores among the three studied classes, suggesting similar academic aptitude. The reported average MCAT score for the classes of 2026, 2027, and 2028 is 513, unchanged from each class year, while the reported undergraduate science GPA for the classes of 2026, 2027, and 2028 are 3.80, 3.81, and 3.82 respectively. Nonetheless, unmeasured variables such as instructor variability or minor changes in assessment design may have influenced performance outcomes. An additional consideration is that while the curriculum of Creighton Health Sciences Campus follows that of the Omaha (main) campus, it has undergone modifications over the subsequent years since its inception. Curricular or faculty changes over time may also have contributed to performance variability.

Third, the reported data showcases the mean scores for quizzes, final exams, and overall block grades. Means of quantitative data sets have the potential to be impacted by outliers in the data set, where extremes of high scores and extremes of low scores could skew the data in their particular direction. The authors acknowledge this potential effect within the data sample and understand that for current claims of NPT having a neutral and/or positive impact on pre-clinical academic performance, a larger data set must be collected to further support the claim.

Lastly, while NPT provides substantial benefits, it is not intended to replace faculty instruction. The absence of formal oversight introduces potential variability in content quality and accuracy [16]. To mitigate this, future iterations of SI should incorporate standardized content templates, and emphasize collaborative faculty support to promote and enhance the SI program.

### Future Directions

To further optimize near-peer education, institutions may consider standardized peer teaching, implementing dedicated tutoring office hours, and expanding SI to include board-style review sessions. Prospective, mixed-methods evaluations could assess not only performance outcomes but also student confidence, sense of belonging, and perceived preparedness for clerkships. Additionally, longitudinal follow-up would help determine whether early participation in NPT influences later clinical performance or teaching competence.

## Conclusions

Informal near-peer teaching, when intentionally integrated into the preclinical curriculum, is associated with neutral and/or positive measurable improvements in academic performance. The SI model at Creighton demonstrates how peer-led instruction can effectively support comprehension in both content-heavy and conceptually challenging courses. These findings underscore the value of NPT as a complementary, scalable, and learner-centered approach to medical education. Continued refinement and structured evaluation of such programs will be essential to ensure they remain evidence-based, high-quality, and sustainable components of medical training.

## Data Availability

The datasets generated for the current study are not publicly available but are available from the corresponding author on reasonable request.
